# AdipoR1 and AdipoR2 maintain membrane fluidity in most human cell types and independently of adiponectin[S]

**DOI:** 10.1194/jlr.M092494

**Published:** 2019-03-19

**Authors:** Mario Ruiz, Marcus Ståhlman, Jan Borén, Marc Pilon

**Affiliations:** Department of Chemistry and Molecular Biology;* University of Gothenburg, Gothenburg, Sweden; Department of Molecular and Clinical Medicine/Wallenberg Laboratory† Institute of Medicine, University of Gothenburg, Gothenburg, Sweden

**Keywords:** fatty acids, desaturases, phospholipids, metabolism, lipotoxicity, receptors, plasma membrane, palmitate

## Abstract

The FA composition of phospholipids must be tightly regulated to maintain optimal cell membrane properties and compensate for a highly variable supply of dietary FAs. Previous studies have shown that AdipoR2 and its homologue PAQR-2 are important regulators of phospholipid FA composition in HEK293 cells and *Caenorhabditis* *elegans*, respectively. Here we show that both AdipoR1 and AdipoR2 are essential for sustaining desaturase expression and high levels of unsaturated FAs in membrane phospholipids of many human cell types, including primary human umbilical vein endothelial cells, and for preventing membrane rigidification in cells challenged with exogenous palmitate, a saturated FA. Three independent methods confirm the role of the AdipoRs as regulators of membrane composition and fluidity: fluorescence recovery after photobleaching, measurements of Laurdan dye generalized polarization, and mass spectrometry to determine the FA composition of phospholipids. Furthermore, we show that the AdipoRs can prevent lipotoxicity in the complete absence of adiponectin, their putative ligand. We propose that the primary cellular function of AdipoR1 and AdipoR2 is to maintain membrane fluidity in most human cell types and that adiponectin is not required for this function.

The AdipoR1 and AdipoR2 proteins were initially identified as putative adiponectin receptors using fluorescent-labeled recombinant adiponectin as bait when screening a cDNA library expressed in Ba/F3 murine cells (1). These proteins are expressed in most/all tissues, localize to the plasma membrane, and contain seven transmembrane domains oriented such that their N terminus is cytosolic and their C terminus is extracellular (1). Since their discovery, several high-profile reports using AdipoR1/2 single- or double-knockout mice have shown that the AdipoRs regulate metabolism and in particular may improve insulin response and generally protect against metabolic syndrome complications, especially during high-fat diet challenges (2–4). However, other reports have not reproduced these findings. In particular, one study found that AdipoR1 and AdipoR2 may have opposite effects on metabolism (5), another showed that the double mutants are embryonic lethal (6), and other studies noted specific defects only in the retina of AdipoR1 mutant mice (7, 8). Finally, a careful study of recombinant adiponectin, which is often used as a means of activating the AdipoRs in published experiments, suggested that any detected biological effect may be caused by the presence of contaminants such as lipopolysaccharide (9), which adds to the confusion regarding the AdipoR literature.

More recently, the crystal structure of the AdipoRs has been solved and suggests that they may be hydrolases: a cavity opening toward the cytoplasm is a likely site for substrate entry and product exit (10). By homology with a yeast homologue, Izh2 (11, 12), the AdipoRs were proposed to act as ceramidases (13, 14). However, the ceramidase activity is at best a slow reaction (15), and it is possible that the AdipoRs may have other substrate specificities. Nevertheless, that the AdipoRs are hydrolases of some sort seems very likely because that is a conserved activity of the large CREST family of related proteins that include the AdipoRs as well as phospholipases such as the yeast Per1p, which has phospholipase A2 activity (16).

Several years ago, we began studying the *Caenorhabditis* *elegans* homologues of the AdipoRs with the hope that leveraging forward genetics in this model organism would lead to new, unbiased insights into their functions. Our findings so far may be briefly summarized as follows: *1*) *C. elegans* has two AdipoR homologues, PAQR-1 and PAQR-2 (17), *2*) worms lacking PAQR-2 show a morphology defect in the thin membranous tail tip and are intolerant to cold (15°C) or to saturated FA (SFA)-rich diets (17–19), *3*) the primary function of PAQR-2 is to maintain membrane fluidity in response to membrane-rigidifying challenges such as cold or SFA-rich diets (18–20), *4*) PAQR-2 function depends on a dedicated protein partner, the single-pass transmembrane protein IGLR-2, with which it colocalizes (18), *5*) PAQR-2 can maintain systemic cell membrane homeostasis nonautonomously via lipid exchange among *C. elegans* tissues (21), and *6*) PAQR-2-deficient worms can be chemically rescued by small amounts of detergents or genetically rescued by mutations that promote FA desaturation or that increase the levels of phospholipids containing long-chain PUFAs (LCPUFAs) (20, 22). It is also important to note that no *C. elegans* homologue of adiponectin has yet been identified. In addition, after several screens for *paqr-2* mutant suppressors or genocopiers, we have found no evidence that PAQR-2 depends on a ligand for its function.

We recently found that several of our observations in *C. elegans* also hold true in mammalian cells. In particular, AdipoR2 is essential in maintaining membrane fluidity in HEK293 cells challenged with the SFA palmitic acid (PA; 16:0); it can also maintain cell-membrane homeostasis nonautonomously (19, 21). Furthermore, and just as in *C. elegans*, HEK293 cells in which AdipoR2 has been knocked down can be rescued by providing LCPUFAs exogenously or by inhibiting the expression of the plasma membrane-localized TLCD1 or TLCD2 proteins (22). The TLCDs function as limiters of LCPUFA incorporation into phospholipids, and their inhibition therefore leads to enhanced incorporation of LCPUFAs, which promotes fluidity (22).

This study focused on further exploring the roles of AdipoR1 and AdipoR2 in regulating membrane homeostasis in human cells. Specifically, we asked whether both AdipoR1 and AdipoR2 act as fluidity regulators and whether many cell types, including primary human cells, require the AdipoRs to regulate membrane fluidity. Finally, we investigated whether the AdipoRs require adiponectin to regulate membrane homeostasis.

## MATERIALS AND METHODS

### Cell culture

HEK293, HepG2, and 1321N1 cells were grown in DMEM containing 1 g/l glucose, pyruvate, and GlutaMAX and supplemented with 10% FBS, 1% nonessential amino acids, 10 mM HEPES, and 1% penicillin and streptomycin (all from Life Technologies) at 37°C in a water-humidified 5% CO_2_ incubator. Cells were subcultured twice a week at 90% confluence. HEK293, HepG2, and 1321N1 cell lines were authenticated by Eurofins. human umbilical vein endothelial cells (HUVECs) (passages 1–5) were obtained from Gibco and cultivated as described previously (23). Briefly, cells were grown in M200 medium (Gibco) containing the Low Serum Growth Supplement (Gibco) and 1% penicillin and streptomycin. Cells were subcultured twice a week at 90% confluence. TrypLE Express reagent (Gibco) was used to detach HUVEC, HEK293, and 1321N1 cells, and Accutase (GE Healthcare) was used to detach HepG2 cells. All cell types were cultivated on treated plastic flasks and multidish plates (Nunc). For microscopy experiments, cells were seeded in glass-bottom dishes (Ibidi) precoated with 0.1% porcine gelatin (Sigma-Aldrich).

### FA treatment

PA, oleic acid, and EPA were dissolved/diluted in sterile DMSO (Sigma-Aldrich) and then mixed with FA-free BSA (Sigma-Aldrich) in serum-free medium for 15 min at room temperature. The molecular ratio of BSA to PA was 1:5.3 in experiments that used 400 μM PA, 1:2.65 in experiments that used 200 μM PA, and 1:0.66 in experiments that used 50 μM PA. Cells were then cultivated in serum-free media containing the FAs for 24 h prior to analysis.

### siRNA treatment

The following predesigned siRNAs were purchased from Dharmacon: AdipoR1 J-007800-10, AdipoR2 J-007801-10, Nontarget D-001810-10, and SCD J-005061-07. HEK293, HepG2, and 1321N1 cell transfection was performed in complete media using 25 nM siRNA and Viromer Blue according to the manufacturer’s instructions 1X (Lipocalyx). HUVECs were transfected using 10 nM siRNA and Lipofectamine RNAiMAX Transfection Reagent following the HUVEC optimized protocol from the manufacturer (Invitrogen). Knockdown gene expression was verified 24 or 48 h after transfection.

### Glucose treatment

Cells were grown in complete media containing 5, 25, or 100 mM glucose (Sigma-Aldich) for 48 h and then switched to serum-free medium supplemented with BSA (0.5%), and keeping the glucose concentration constant at 5, 25, or 100 mM for another 24 h and then analyzed.

### Quantitative PCR

Total cellular RNA was isolated using RNeasy Kit according to the manufacturer’s instructions (Qiagen) and quantified using a NanoDrop spectrophotometer (ND-1000; Thermo Fisher Scientific). cDNA was obtained using a High-Capacity cDNA Reverse Transcription Kit (Applied Biosystems) with random hexamers. Quantitative PCR (qPCR) was performed with a CFX Connect thermal cycler (Bio-Rad) using HOT FIREpol EvaGreen qPCR Supermix (Solis Biodyne) and standard primers. Samples were measured as triplicates. The relative expression of each gene was calculated according to the ΔΔCT method (24). Expression of the housekeeping gene *PPIA* was used to normalize for variations in RNA input. Primers used were as follows: AdipoR1 forward, CCATCTGCTTGGTTTCGTGC; AdipoR1 reverse, AGACGGTGTGAAAGAGCCAG; AdipoR2 forward, TCATCTGTGTGC­TGGGCATT; Adipo2 reverse, CTATCTGCCCTATGGTGGCG; PPIA forward, GTCTCCTTTGAGCTGTTTGCAG; PPIA reverse, GGACAAGATGCCAGGACCC; SCD forward, TTCGTTGCCACTTTCTTGCG; SCD reverse, TGGTGGTAGTTGTGGAAGCC; FADS1 forward, TGGCTAGTGATCGACCGTAA; FADS1 reverse, GGCCCTTGTTGATGTGGAAG; FADS2 forward, GGGCCGTCAGCTACTACATC; and FADS2 reverse, ACAAACCAGTGGCTCTCCAG.

qPCR for adiponectin was executed on a QuantStudio7 Flex Real-Time PCR System thermal cycler using Power SYBR Green PCR Master Mix (Applied Biosystems). Two sets of primers for adiponectin were used: *1*) adiponectin forward (AGGGCATCCGGGCCATAAT) and reverse (CTCCGGTTTCACCGATGTCT) and *2*) adiponectin forward (TGGTGAGAAGGGTGAGAAAGG) and reverse (CTCCAATCCCACACTGAATGC), respectively. The second set of adiponectin primers was shared by Matthew Harms. cDNA from human subcutaneous abdominal adipose tissue and human breast adipose tissue was a gift from Xiao-Rong Peng and Henrik Palmgren (AstraZeneca); samples of adipose tissues were collected from patients undergoing elective surgery at Sahlgrenska University Hospital, Gothenburg, Sweden. All study subjects received written and oral information before giving written informed consent for the use of the tissue. The studies abide by the Declaration of Helsinki principles and were approved by the regional ethical review board in Gothenburg, Sweden.

### Protein samples and Western blot

Cellular proteins were extracted using a lysis buffer (1% Nonidet P-40, 0.1% SDS, 10% glycerol, 1% sodium deoxycholate, 1 mM DTT, 1 mM EDTA, 100 mM HEPES, 100 mM KCl) containing Halt Protease Inhibitor Cocktail (1×; Pierce) on ice for 10 min. Upon lysis completion, cell lysates were centrifuged at 13,000 rpm for 10 min at 4°C. The soluble fraction was kept for further analysis, and the protein sample concentration was quantified using the BCA protein assay kit (Pierce) according to the manufacturer’s instructions. Twenty micrograms of protein were mixed with Laemmli sample loading buffer (Bio-Rad), heated to 37°C for 10 min, and loaded in 4% to 15% gradient precast SDS gels (Bio-Rad). After electrophoresis, the proteins were transferred to nitrocellulose membranes using Trans-Blot Turbo Transfer Packs and a Trans Blot Turbo apparatus/predefined mixed-MW program (Bio-Rad). Blots were blocked with 5% nonfat dry milk in PBS for 1 h at room temperature. Blots were incubated with anti-human AdipoR1 rabbit IgG at 1 µg/ml in 5% nonfat dry milk in PBS with Tween-20 (PBS-T; 0.05% Tween-20) overnight at 4°C following previously published recommendations (8). Blots were then washed with PBS-T and incubated with a swine anti-rabbit IgG/HRP (1:3000 dilution; Dako) and washed again with PBS-T. Blots were developed with ECL (Immobilon Western; Millipore), and the signal was visualized with a digital camera (VersaDoc; Bio-Rad). Blots were then stripped and reprobed with anti-GAPDH (14C10) rabbit IgG (1:2500 dilution; Cell Signaling). PageRuler Plus prestained protein ladder was used to assess molecular weight (Thermo Fisher Scientific). Western blots were quantified by densitometry using Image Lab version 6 software.

### Lipidomics

Samples were prepared as previously described (19, 22). Briefly, cells were cultivated in serum-free media with or without FAs for 24 h prior to harvesting using TrypLE Express (Gibco). For lipid extraction, the pellet was sonicated for 10 min in methanol and then extracted according to published methods (25). Internal standards were added during the extraction. Lipid extracts were evaporated and reconstituted in chloroform-methanol (1:2) with 5 mM ammonium acetate. This solution was infused directly (shotgun approach) into a QTRAP 5500 mass spectrophotometer (Sciex) equipped with a TriVersa NanoMate (Advion Bioscience) as described previously (26). Phospholipids were measured using multiple precursor ion scanning, which gathers information about individual phospholipids by scoring many FA fragments in the Q3 of the mass spectrometer (27, 28), and triacylglycerols (TAGs) were measured using neutral-loss scanning (29). The data were evaluated using the LipidView software (Sciex). The complete lipidomics data set is provided in the supplemental Table S1.

### FRAP

Live HEK293 cells were stained with BODIPY 500/510 C1, C12 (Invitrogen). Fluorescence recovery after photobleaching (FRAP) images were acquired with an LSM880 confocal microscope equipped with a live cell chamber (set at 37° and 5% CO_2_) and ZEN software (Zeiss) with a 40× water-immersion objective as previously described (19, 21, 22).

### Laurdan dye measurement of membrane fluidity

Live cells were stained with Laurdan dye (6-dodecanoyl-2-dimethylaminonaphthalene) (Thermo Fisher Scientific) at 15 μM (HEK293 and HepG2 cells) or 10 μM (HUVECs) for 45 min. Images were acquired with an LSM880 confocal microscope equipped with a live cell chamber (set at 37° and 5% CO_2_) and ZEN software with a 40× water-immersion objective as described previously (21). Cells were excited with a 405 nm laser, and the emission was recorded between 410 and 461 nm (ordered phase) and between 470 and 530 nm (disordered phase). Pictures were acquired with 16 bit image depth and 1,024 × 1,024 resolution using a pixel dwell of ∼1.02 μs. Images were analyzed using ImageJ version 1.47 software (30) following published guidelines (31).

### Trypan blue staining

After 24 h of treatment, cell supernatant was collected, and cells were detached and mixed again with their respective supernatant. The cell suspension was then mixed 1:1 with a 0.4% trypan blue solution (Gibco) and loaded in a hemacytometer and examined immediately under the microscope. The percentage of positive and negative cells in at least four quadrants was registered.

### Adiponectin (or AdipoQ) treatment

Recombinant full-length adiponectin (produced in HEK293 cells and forming high-molecular-weight and hexameric species) was purchased from Enzo and used at 5 µg/ml (14) or 10 µg/ml. Cells were then cultivated in serum-free media containing adiponectin and 400 μM PA for 24 h prior to analysis.

### Statistics

*t*-Tests were used to identify significant differences between treatments/genotypes. Error bars show the SDs in histograms and SEMs in FRAP curves. Asterisks and hash signs are used in the figures to indicate various degrees of significance.

## RESULTS

### Both AdipoR1 and AdipoR2 regulate membrane fluidity in HEK293 cells

**Fig. 1.**
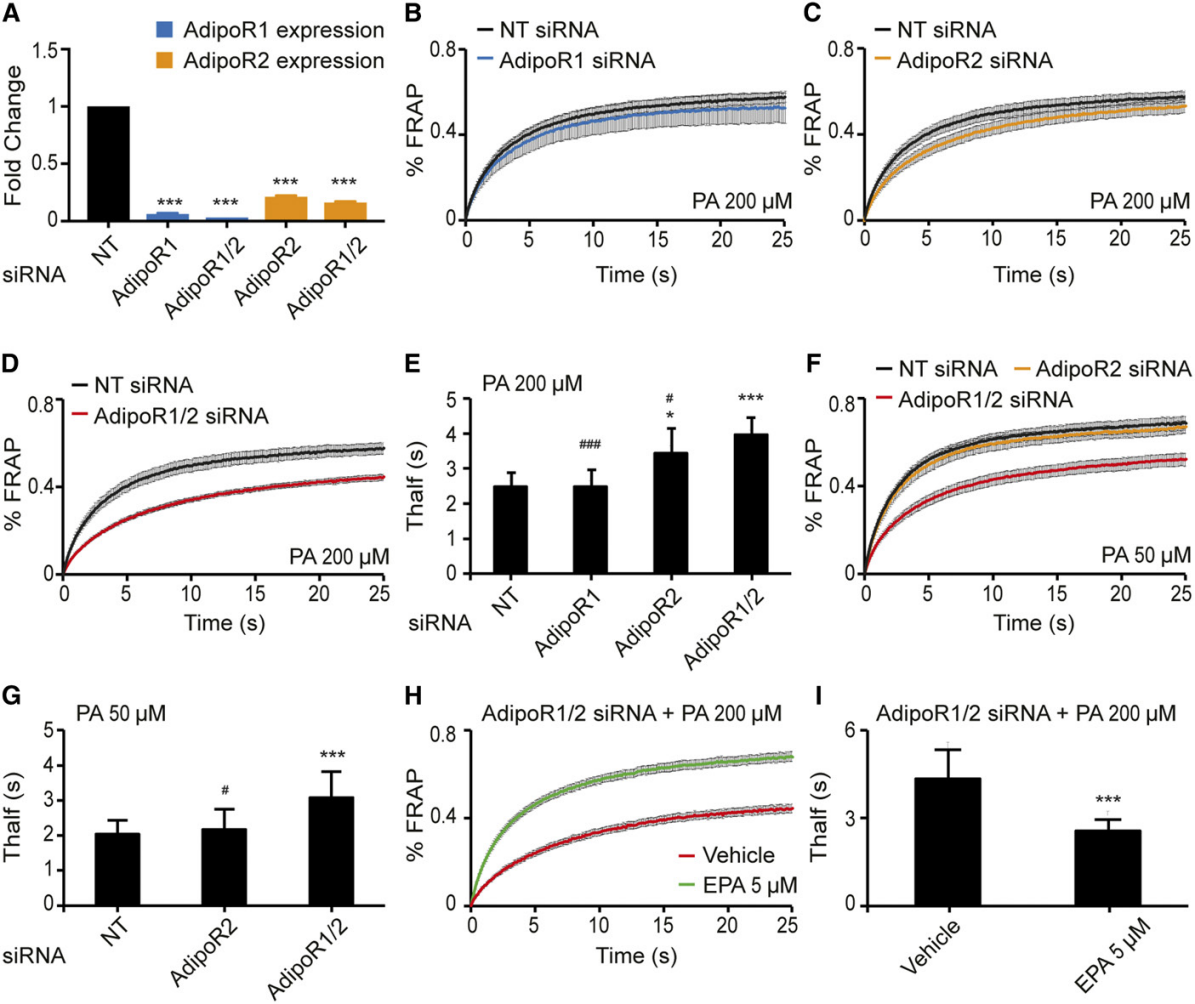
FRAP analysis showing that AdipoR1 and AdipoR2 redundantly maintain membrane fluidity in HEK293 cells. A: qPCR results showing the efficiency of the knockdown using NT, AdipoR1, and/or AdipoR2 siRNA. The expression levels are normalized to the NT value. B–D: FRAP results in HEK293 cells challenged with 200 μM PA and treated with NT, AdipoR1, AdipoR2, or AdipoR1/2 siRNA (*n* = 6–14). E: Average *T_half_* values (the time by which half of the maximum fluorescence recovery is reached) from multiple experiments as in panels B–D. F: FRAP results in HEK293 cells challenged with 50 μM PA and treated with NT, AdipoR2, or AdipoR1/2 siRNA (*n* = 10–13). G: Average *T_half_* values from panel F. H, I: FRAP results in HEK293 cells challenged with 200 μM PA and treated with either vehicle (DMSO) or 5 μM EPA (*n* = 10). Error bars show the SDs in histograms and SEMs in FRAP panels. *P < 0.05 and ***P < 0.001 compared with the control treatment. #*P* < 0.05 and ###*P* < 0.001 compared with the AdipoR1/2 siRNA treatment. NT, nontarget.

We used siRNA to efficiently knock down the expression of AdipoR1 and AdipoR2 singly or simultaneously in HEK293 cells: qPCR confirmed the knockdown of the transcripts (**Fig. 1A**) , and a Western blot confirmed protein downregulation for AdipoR1 (supplemental Fig. S1A). We could not obtain a useful antibody for AdipoR2. Knockdown of AdipoR1 and/or AdipoR2 had no effect on membrane fluidity of the cells under basal culture condition as determined using FRAP (supplemental Fig. S1B–E). Note that the FRAP method measures the lateral diffusion rate of the BODIPY-C12 fluorophore used in these experiments; throughout this article we use the term “membrane fluidity” as a proxy for what is likely a complex phenomenon reflecting several distinct membrane biophysical properties such as fluidity, phase behavior, thickness, or compressibility (32–34). siRNA knockdown of AdipoR1 also had little effect on the membrane fluidity of HEK293 cells challenged with 200 μM PA, but inhibiting AdipoR2 caused a clear loss of membrane fluidity under these conditions (Fig. 1B, C), while inhibiting both AdipoR1 and AdipoR2 had the most dramatic effect (Fig. 1D, E). These results demonstrate that AdipoR2 is more important than AdipoR1 in protecting against the rigidifying effects of PA but that AdipoR1 also contributes to the maintenance of membrane fluidity. In other words, AdipoR1 and AdipoR2 have partially redundant functions in membrane homeostasis. Most studies of the effects of PA on cells require relatively high concentrations to elicit a detectable effect, with 400 μM being a typical concentration. The pronounced sensitivity of the double AdipoR1/2 siRNA-treated cells to 200 μM PA suggest that these proteins are a primary mechanism protecting cells against PA lipotoxicity. Indeed, HEK293 cells treated with double AdipoR1/2 siRNA show a dramatic loss of membrane fluidity even when challenged with as little as 50 μM PA (Fig. 1F, G), which is exceptionally low compared with other published studies. Note that AdipoR2 knockdown causes membrane rigidification when cells are challenged with 200 μM palmitate but not when treated with vehicle alone. In addition, this palmitate-induced rigidification in AdipoR2 knockdown cells can be prevented by including equimolar amounts of the MUFA oleic acid, suggesting that a function of the AdipoRs during palmitate challenge is to restore SFA/unsaturated FA (UFA) balance (supplemental Fig. S1F–J). The loss of membrane fluidity in AdipoR1/2 siRNA-treated cells challenged with 200 μM PA can also be entirely suppressed by the inclusion of as little as 5 μM of the LCPUFA EPA (20:5) in the culture media (Fig. 1H, I); EPA is a potent fluidizing FA, and its ability to restore membrane fluidity in the AdipoR1/2 siRNA-treated cells is consistent with these proteins playing a role in regulating the FA composition of phospholipids.

### The Laurdan dye method confirms the roles of AdipoR1/2 in membrane homeostasis

**Fig. 2.**
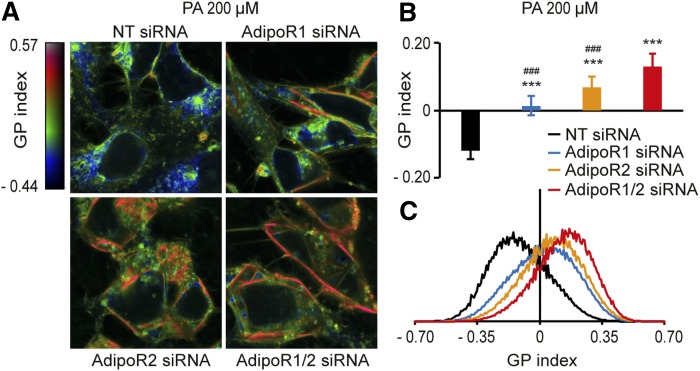
The Laurdan dye method confirms that AdipoR1 and AdipoR2 are required to maintain membrane fluidity in HEK293 cells. A: Pseudocolor images showing the Laurdan dye GP index at each pixel position in HEK293 cells challenged with 200 μM PA and treated with NT, AdipoR1, and/or AdipoR2 siRNA. Note the pronounced rigidification of the plasma membrane in the AdipoR1/2 siRNA-treated cells. B: Average GP index from several images as in panel A (*n* = 10–15). C: Distribution of the GP index values in representative images for each treatment. Error bars show the SDs. ***P < 0.001 compared with the control treatment. ###*P* < 0.001 compared with the AdipoR1/2 siRNA treatment. GP, generalized polarization; NT, nontarget.

Our measurements of membrane fluidity have so far relied heavily on the FRAP method. To guard against any misleading interpretations, it is important to verify critical results with independent methods. Therefore, we also made use of the Laurdan dye method to monitor membrane fluidity. This method relies on the fact that the membrane-bound Laurdan dye emits fluorescent light at different wavelengths when water is present within the phospholipid bilayer, which happens more readily in fluid membranes. This method has the additional advantages that multiple cells are imaged simultaneously, that subcellular regions with increased rigidity can be identified, and that the images can be scored quantitatively using an automated ImageJ script (31). Analysis of membrane fluidity using the Laurdan dye method corroborates the findings using the FRAP method with the exception that it can now detect a role for AdipoR1. Specifically, we found that siRNA knockdown of AdipoR1 or AdipoR2 singly or together leads only to a minor membrane rigidification under basal conditions (supplemental Fig. S1K–M) but that both AdipoR1 and AdipoR2 are required to maintain membrane fluidity when HEK293 cells are challenged with 200 μM PA (**Fig. 2A**–C). Furthermore, inhibiting both simultaneously leads to a much more severe rigidifying effect of PA (Fig. 2A–C), which indicates that AdipoR1 and AdipoR2 have overlapping functions. Also, we noted that the plasma membrane appears to be most affected by rigidification when AdipoR1 and AdipoR2 are inhibited. This is particularly interesting because AdipoR1 and AdipoR2 are localized to the plasma membrane and may have an especially important function in maintaining fluidity in that membrane.

### AdipoR1 and AdipoR2 promote membrane fluidity via several desaturases

**Fig. 3.**
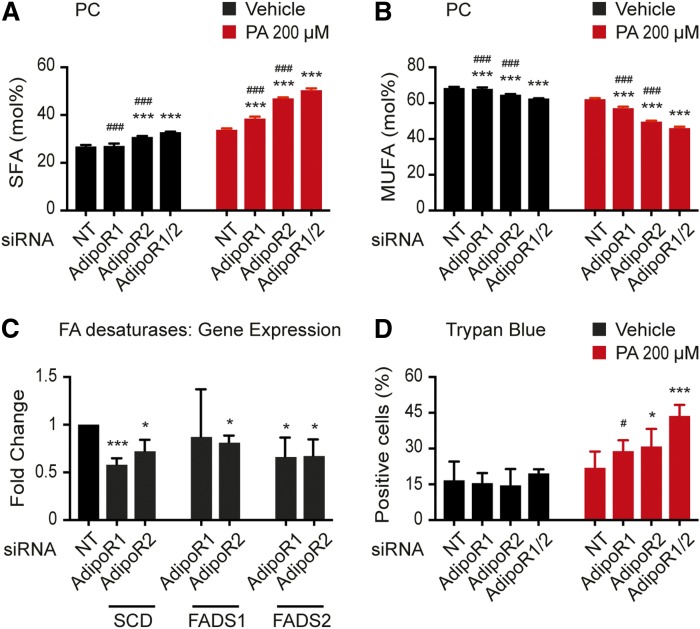
The AdipoRs are required to maintain MUFA levels in PCs, sustain desaturase gene expression, and prevent lipotoxicity by PA. A, B: SFA and MUFA abundance (mol.%) in the PCs of HEK293 cells cultivated in the presence of either vehicle (DMSO) or 200 μM PA and treated with NT, AdipoR1, and/or AdipoR2 siRNA (*n* = 3). C: qPCR results showing the expression of three desaturases in HEK293 cells following knockdown using NT, AdipoR1, and/or AdipoR2 siRNA. The expression levels are normalized to the NT value**. **D: Percentage of dead HEK293 cells (trypan blue-positive) following cultivation in the presence of either vehicle (DMSO) or 200 μM PA and treated with NT, AdipoR1, and/or AdipoR2 siRNA. Error bars show the SDs. *P < 0.05 and ***P < 0.001 compared with the control treatment. #*P* < 0.05 and ###*P* < 0.001 compared with the AdipoR1/2 siRNA treatment. NT, nontarget.

We have previously shown that the *C. elegans paqr-2* mutant has an excessively high SFA-UFA ratio among phospholipids and is unable to stimulate FA desaturation upon membrane-rigidifying challenges (cold or SFA-rich diets). This role in membrane homeostasis is also conserved for AdipoR1 and AdipoR2 in human cells. siRNA against AdipoR1 or AdipoR2 causes HEK293 cells to have excess SFAs in their phosphatidylcholines (PCs) and phosphatidylethanolamines (PEs) both under basal conditions and even more so when challenged with 200 μM PA, and this effect is increased when AdipoR1 and AdipoR2 are simultaneously inhibited (**Fig. 3A**, B, supplemental Fig. S2). The change in FA composition of the phospholipids correlates with reduced desaturase expression: AdipoR1 siRNA-treated cells have reduced expression of SCD and FADS2, while AdipoR2 siRNA-treated cells have reduced expression of SCD, FADS1, and FADS2 (Fig. 3C). Not surprisingly then, knockdown of AdipoR1 and/or AdipoR2 increases the lipotoxicity of PA: while 200 μM palmitate is well-tolerated by HEK293 cells treated with nontarget siRNA, knockdown of the AdipoRs greatly increases the number of dead cells in the presence of PA (but not in basal media; Fig. 3D). Altogether these results indicate that the AdipoRs redundantly maintain membrane fluidity in PA-challenged HEK293 cells by regulating the expression of desaturases and that this activity is essential in preventing lipotoxicity by SFAs. Accordingly, siRNA against AdipoR1 or AdipoR2 also causes HEK293 cells to have excess SFAs in TAGs (supplemental Fig. S2C, D), which again likely reflects decreased desaturase activity.

The *C. elegans paqr-2* mutant, which lacks a functional PAQR-2 protein that is homologous to the AdipoRs, is unable to grow in the presence of glucose, even at concentrations as low as 4 mM. This is because the glucose is converted to SFAs by the dietary *Escherichia coli*, which leads to membrane rigidification in the worms (19). In other words, glucose in itself has no effect on the *paqr-2* mutant. Similarly, glucose has no effect on the membrane fluidity of AdipoR1 and/or AdipoR2 siRNA-treated HEK293 cells, even when applied at concentrations of 100 mM, and it also had no effect on the SFA and MUFA content in the PCs, PEs, or TAGs of cells treated with AdipoR2 siRNA (supplemental Fig. S3). We conclude that, as with the *C. elegans* PAQR-2, the AdipoRs are required specifically to respond to the rigidifying challenge posed by exogenous SFAs and not for glucose tolerance.

### AdipoR1 and AdipoR2 maintain membrane fluidity in several cell types

**Fig. 4.**
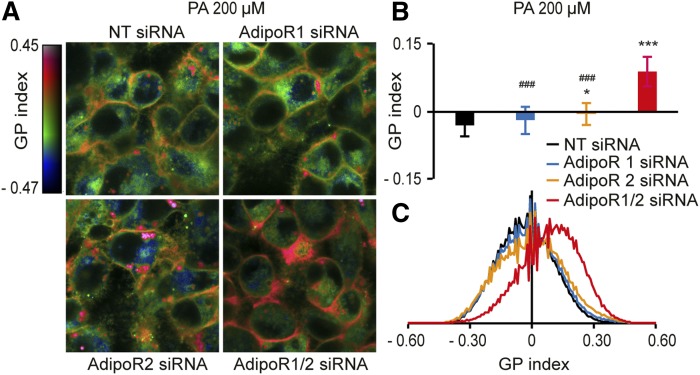
The Laurdan dye method shows that AdipoR1 and AdipoR2 are required to maintain membrane fluidity in HepG2 cells. A: Pseudocolor images showing the Laurdan dye GP index at each pixel position in HepG2 cells challenged with 200 μM PA and treated with NT, AdipoR1, and/or AdipoR2 siRNA. Note the pronounced rigidification of the plasma membrane in the AdipoR1/2 siRNA-treated cells. B: Average GP index from several images as in panel A (*n* = 15). C: Distribution of the GP index values in representative images for each treatment. Error bars show the SDs. *P < 0.05 and ***P < 0.001 compared with the control treatment. #*P* < 0.05 and ###*P* < 0.001 compared with the AdipoR1/2 siRNA treatment. GP, generalized polarization; NT, nontarget.

The AdipoRs are widely expressed, and it is therefore possible that they are important regulators of membrane fluidity in many, and perhaps even most, cell types. To explore this possibility, we investigated the effect of AdipoR1 and/or AdipoR2 siRNA on hepatocyte-derived HepG2 cells and astrocyte-like 1321N1 cells. siRNA knockdown in HepG2 cells reduced AdipoR1 mRNA levels by nearly 90% and AdipoR2 levels by more than 50% (supplemental Fig. S4A); siRNA knockdown of AdipoR1 caused a ∼60% reduction in protein levels (supplemental Fig. S4B, C). Knockdown of AdipoR1 alone had little effect on the membrane fluidity (measured with the Laurdan dye method) of HepG2 cells in basal media or media supplemented with 200 μM PA (**Fig. 4A**–C, supplemental Fig. S4D–F). In contrast, AdipoR2 knockdown caused a reduced membrane fluidity both in basal and PA-supplemented media, and this effect was greatly increased when AdipoR1 and AdipoR2 were simultaneously knocked down (Fig. 4A–C, supplemental Fig. S4D–F). As in HEK293 cells, knockdown of AdipoR1 and/or AdipoR2 resulted in the loss of cell viability upon PA challenge (supplemental Fig. S4G) and an increase in SFAs at the expense of MUFAs in PCs, PEs, and TAGs, especially when the cells were challenged with PA (supplemental Fig. S5H–M). Very similar results were obtained with the 1321N1 cell line (supplemental Fig. S4N–U). In summary, these results suggest that AdipoR1 and AdipoR2 are redundantly essential in maintaining membrane fluidity in all three cell lines tested, each representing a different type of cell, namely embryonic kidney cells, hepatocytes, and astrocytes. Maintaining membrane fluidity is likely the fundamental function of the AdipoRs and is particularly required upon membrane-rigidifying challenges such as when SFAs are provided exogenously.

### The AdipoRs maintain membrane fluidity in human primary cells independently of adiponectin

**Fig. 5.**
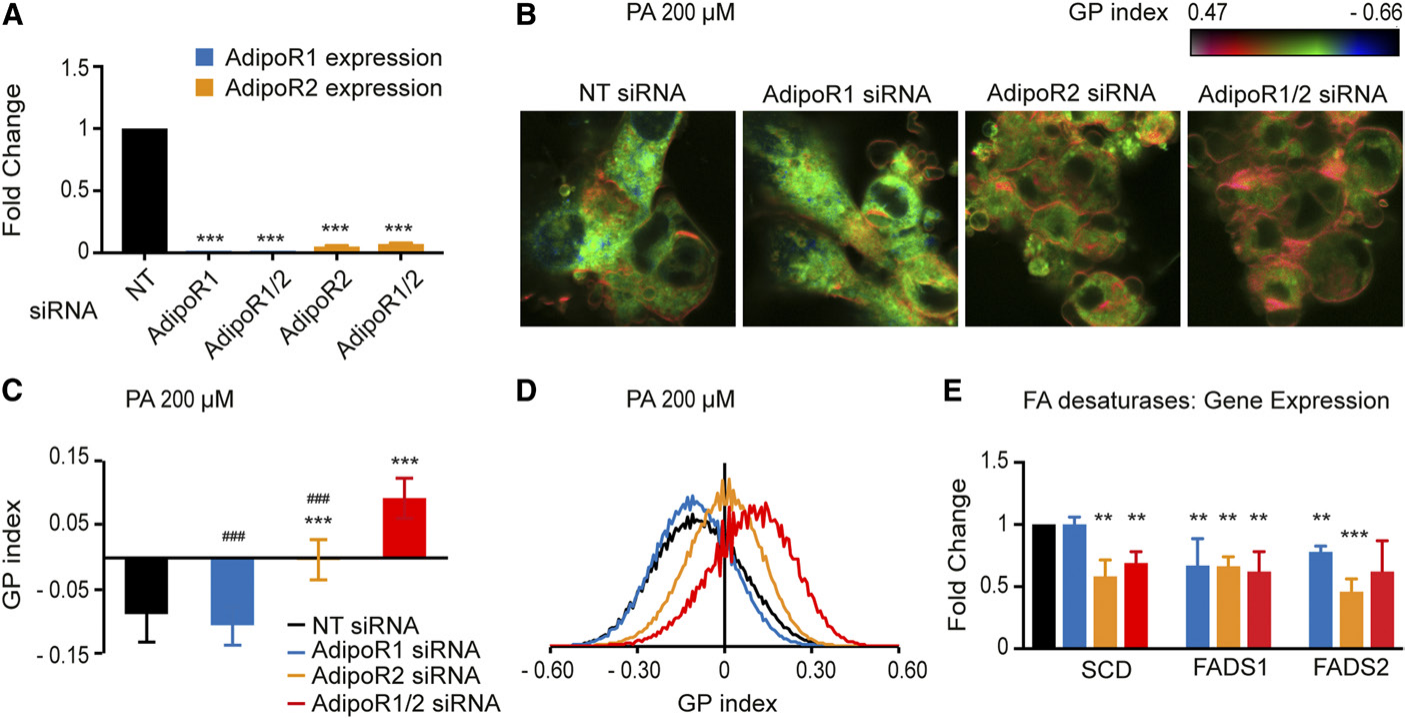
The AdipoRs maintain membrane fluidity and desaturase expression in primary human cells (HUVECs). A: qPCR results showing the efficiency of the knockdown in HUVECs using NT, AdipoR1, and/or AdipoR2 siRNA. The expression levels are normalized to the NT value. B: Pseudocolor images showing the Laurdan dye GP index at each pixel position in HUVECs challenged with 200 μM PA and treated with NT, AdipoR1, and/or AdipoR2 siRNA. Note the pronounced rigidification of the plasma membrane in the AdipoR1/2 siRNA-treated cells. C: Average GP index from several images as in panel A (*n* = 10–15). D: Distribution of the GP index values in representative images for each treatment. E: qPCR results showing the expression of three desaturases in HEK293 cells following knockdown using NT, AdipoR1, and/or AdipoR2 siRNA. The expression levels are normalized to the NT value. Error bars show the SDs. **P < 0.01 and ***P < 0.001 compared with the control treatment. ###*P* < 0.001 compared with the AdipoR1/2 siRNA treatment. GP, generalized polarization; NT, nontarget.

Established cell lines harbor a multitude of mutations and are abnormal in many ways, including in lipid metabolism. It was therefore important to verify our key findings in human primary cells. We began by optimizing knockdown of AdipoR1 and/or AdipoR2 in HUVECs, being able to inhibit >90% of their expression (**Fig. 5A**). We then also optimized the Laurdan dye method for HUVECs and found that it clearly detected membrane rigidification when these cells were challenged with 400 μM PA (supplemental Fig. S5A–C), which is the concentration most often used to rigidify cell membranes in control cells. Knockdown of AdipoR1 alone had little effect on the HUVECs challenged with 200 μM PA (Fig. 5B–D). In contrast, AdipoR2 knockdown caused a reduced membrane fluidity in 200 μM PA-supplemented media, and this effect was greatly increased when AdipoR1 and AdipoR2 were simultaneously knocked down (Fig. 5B–D). Knockdown of AdipoR1 and/or AdipoR2 had no effect on the membrane fluidity of HUVECs grown in basal media (supplemental Fig. S5D–F). We conclude that AdipoR1 and AdipoR2 are essential in maintaining membrane fluidity in primary human endothelial cells, especially in the presence of exogenous SFAs.

As with the HEK293 cells, inhibition of either AdipoR1 and AdipoR2 led to reduced expression of the FA desaturases SCD, FADS1, and FADS2 (only SCD was not downregulated by AdipoR1 siRNA; Fig. 5E). Desaturase activity is essential in maintaining membrane fluidity upon a PA challenge. In particular, SCD inhibition by siRNA (supplemental Fig. S5G) led to decreased membrane fluidity of HUVECs incubated with 200 μM PA, but not in basal media (supplemental Fig. S5H–M).

Finally, we note that adiponectin, a proposed ligand for the AdipoRs, was never included in any of our experiments. Indeed, most experiments involved long periods of incubations in serum-free media (basal) or serum-free media supplemented with PA, and there is likely no expression of adiponectin from the tested cells themselves because this is an adipocyte-specific protein. As a definitive test we performed qPCR analysis using mRNA from the three studied cell lines (HEK293, HepG2, and 1321N1), HUVECs and human adipose tissue. Using this assay, we could detect strong adiponectin expression in the adipocyte sample and no expression in the cell lines or HUVECs; the house-keeping control gene *PPIA* was detected in all samples (**Table 1**, supplemental Fig. S6A). Supplementing HEK293 cells with 5 μg/ml (14) or 10 μg/ml recombinant adiponectin (produced from a mammalian cell-expression system) did not improve their ability to prevent membrane rigidification by 400 μM PA (the lowest concentration that reduced fluidity in control HEK293 cells), suggesting that adiponectin does not potentiate the activity of the AdipoRs, at least in this context (supplemental Fig. S6B, C). We conclude from this that adiponectin is not required for the ability of the AdipoRs to act as regulators of membrane fluidity in human cells.

**TABLE 1.** qPCR results for the detection of adiponectin (primer set I) and PPIA expression in all cell types studied

Sample	Adiponectin	PPIA
Subcutaneous fat	17.01	20.47
Breast fat	16.76	19.91
HUVEC 1	Nondetected	15.13
HUVEC 2	Nondetected	16.75
HUVEC 3	Nondetected	17.82
HEK293 1	Nondetected	15.80
HEK293 2	Nondetected	15.96
HepG2	Nondetected	16.98
1321N1 1	Nondetected	15.30
1321N1 2	Nondetected	15.19
Nontemplate control	Nondetected	34.63

The CT values are indicated for each sample/gene. Results from different cDNA preparations are shown for several samples (numbered 1, 2, or 3).

## DISCUSSION

**Fig. 6.**
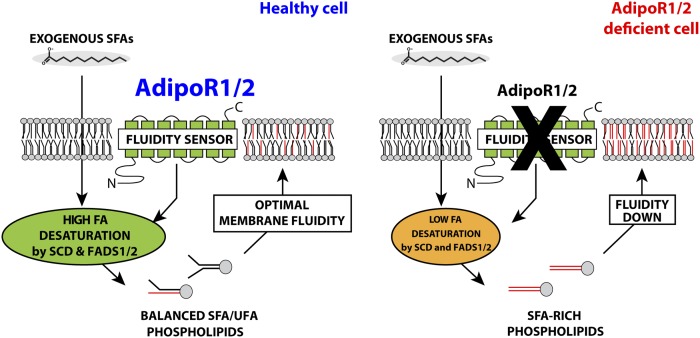
Model for the role of the AdipoRs in membrane homeostasis. In healthy cells challenged with exogenous SFAs, the AdipoRs sense membrane rigidification and signals to promote desaturase enzyme expression, resulting in increased incorporation of fluidizing UFAs into phospholipids. AdipoR-deficient cells fail to promote desaturase activity to compensate for the exogenously provided SFAs that become incorporated into phospholipids, leading to membrane rigidification and hence lipotoxicity.

The three most important conclusions from the present work are *1*) both AdipoR1 and AdipoR2 maintain membrane fluidity by promoting desaturase activity and, hence, MUFA levels in phospholipids; *2*) AdipoR1 and AdipoR2 help maintain membrane fluidity in multiple cell types, including primary human endothelial cells; and *3*) adiponectin is not required for the function of the AdipoRs as fluidity regulators. These conclusions lead to a simple model of AdipoR function (**Fig. 6**) and will now be discussed in the broader context of existing literature.

That both AdipoR1 and AdipoR2 have similar functions is not surprising given their high degree of amino acid identity [∼66% (1)]. Many tissues express both proteins, and it is likely that many cell types also express both, as was the case for all four cell types studied here (HEK293, HepG2, 1321N1, and primary HUVECs). Coexpression of two proteins with similar functions provides redundancy, and hence robustness, in the maintenance of membrane homeostasis. We note, however, that in all cell types it was the inhibition of AdipoR2 that had the strongest effect on membrane fluidity, suggesting that AdipoR2 plays a larger role than AdipoR1. This is interesting because the AdipoR2 knockout mice are reported to have a more severe phenotype, including an enlarged brain, male sterility, low weight, and increased risk of diabetes (5, 35), than the AdipoR1 mice, which primarily show a retina defect that is also found in humans with mutations in AdipoR1 (7, 8, 36, 37), as well as a tendency to gain weight (5). That the two genes have overlapping functions is clear from the fact that double-mutant mice lacking both AdipoR1 and AdipoR2 have more severe metabolic defects than either single mutants (2) and may even be embryonic lethal according to another study (6). The situation in mice therefore echoes that in *C. elegans*, in which the two AdipoR homologues, *paqr-1* and *paqr-2*, are also partially redundant (the double mutant is more severe than the single mutant), although *paqr-2* is clearly more important because it shows strong phenotypes as a single mutant (cold and SFA intolerance and a morphology defect in the thin membranous tail tip) that are accompanied by the loss of membrane fluidity (17).

The knowledge that AdipoR1 and AdipoR2 are important for membrane homeostasis, primarily promoting FA desaturation to restore fluidity, may lead to a better understanding of several of the mouse mutant phenotypes. For example, the enlarged brain of the AdipoR2 knockout mice may be secondary to an inability to sustain membrane fluidity in the mutant (5). The brain is exceedingly dependent on membrane fluidity for the process of neurotransmitter vesicle trafficking and fusion at the synapses (38). Similarly, spermatogenesis is critically dependent on an abundance of fluid membrane, and desaturase mutants are therefore sterile (39). It will be interesting to revisit these and other AdipoR2 knockout mouse phenotypes and test the specific hypothesis that they are the result of failures in membrane fluidity homeostasis. Further, several articles have implicated the AdipoRs as being important in preventing metabolic syndrome complications, for example, when mice are fed high-fat diets. Given the extensive literature documenting high SFA content and membrane rigidity in diabetics (40, 41), it will be very interesting to see whether the alleged protective effects of the AdipoRs may be explained precisely because they help delay or even prevent such membrane rigidification. In particular, pancreatic β-cell function depends on delicate membrane trafficking and fusion events essential for insulin secretion (42). Such processes are very sensitive to membrane rigidification, and the maintenance of membrane fluidity in β-cells may be an important function of the AdipoRs in the context of the metabolic syndrome.

The AdipoRs were initially identified as adiponectin receptors in a cDNA expression screen to identify clones that would bind fluorescently labeled recombinant adiponectin (1). Several studies later suggested that the activity of the AdipoRs, for example in preventing metabolic defects upon high-fat diets in mice, were dependent on adiponectin, usually provided in its recombinant, *E. coli*-expressed form (2). The functionality of recombinant adiponectin has, however, been questioned (9), and the hypothesis that adiponectin could act as a hormone may be questioned on the grounds that it is simply too abundant a serum protein to serve such a function (adiponectin is present at μg/ml levels compared with other hormones such as insulin or leptin that are present at ng/ml levels), although it is possible that much less abundant multimeric forms with dynamically regulated levels are the true nature of adiponectin as a hormone (43, 44). In any case, the three different cell lines and human primary cells studied here do not express adiponectin, and yet we could demonstrate clear functions for both AdipoR1 and AdipoR2 in preventing membrane rigidification by PA; the addition of recombinant adiponectin also did not improve the ability of HEK293 cells to maintain membrane fluidity when challenged with palmitate, suggesting that it is not limiting for AdipoR function in these assays. This again echoes the situation in *C. elegans*, in which no adiponectin homologue has so far been identified and in which several forward genetic screens for suppressors or genocopiers of *paqr-2* have failed to identify a putative ligand (17, 20, 22, 45).

One limitation of our experiments is that we do not know the extent of protein activity reduction achieved by the siRNA treatments. Good antibodies to these proteins have been difficult to produce. For AdipoR1, more than 15 different antibodies were recently rigorously tested, and a single one was found to specifically recognize AdipoR1 in a useful way (8). Using this antibody in Western blot shows a >90% and ∼50% reduction in AdipoR1 levels in siRNA-treated HEK293 and HepG2 cells, respectively (supplemental Figs. S1A, S4B**)**. We also tried two different AdipoR2 antibodies without success: both produced a very high level of background bands. This likely explains why AdipoR2 antibodies have so rarely been used in studies to detect the endogenous protein. However, we are confident in the siRNA successfully inhibiting AdipoR2 in our experiments for several reasons: *1*) two different siRNA oligo pairs produce the same membrane rigidification effect (19); *2*) the qPCR clearly shows efficient downregulation of the AdipoR2 mRNA in cell lines and primary cells (Figs. 3C, 5E); *3*) the effects of siRNA against AdipoR2 are additive with that of AdipoR1 knockdown; and *4*) the membrane rigidification caused by siRNA against AdipoR2 is accompanied by excess SFAs in phospholipids in all cell types tested, just as it is in *C. elegans* (19, 20).

Finally, it is interesting to note that the methods used to evaluate membrane properties (FRAP and Laurdan dye fluorescence) and composition (lipidomics) did not distinguish among the various organellar membranes of the cells examined. Rather, all three methods scored the entire cells. Of the three methods, it is the Laurdan dye fluorescence that provides the best spatial resolution. An examination of the generalized polarization index pseudocolor images (e.g., Fig. 2A for HEK293 cells, Fig. 4A for HepG2 cells, and Fig. 5B for HUVECs) indicates membrane rigidification across the entire cells but clearly most pronounced in the plasma membranes. This is interesting because the AdipoRs are the only sense-and-response membrane sensors localized to the plasma membrane and may therefore be especially important for plasma membrane homeostasis. The AdipoRs may therefore complement the activities of other guardians of cellular membranes, such as PCYT1A and Tafazzin [sense and repair packing defects in the inner nuclear membrane and mitochondria, respectively (46, 47)], IRE1 [senses bilayer stress in the ER (48)], or the SREBPs (sense cholesterol and PC levels (49, 50)]. It will be interesting in the future to perform cell-fractionation experiments in AdipoR-deficient cells to better understand the organelle-specific roles of these proteins.

In conclusion, the present findings indicate that the AdipoRs are important regulators of membrane fluidity in most cell types, including human primary endothelial cells, and that they can carry out this function without any ligand being present.

## Supplementary Material

Supplemental Data
