# Supplementary material for: AdipoR1 and AdipoR2 maintain membrane fluidity in most human cell types and independently of adiponectin
Source: J Lipid Res. 2019 Mar 19;60(5):995–1004. doi: 10.1194/jlr.M092494 (PMC6495173; doi:10.1194/jlr.M092494)
Supplement: Supplemental Data [file supp_60_5_995__index.html]

AdipoR1 and AdipoR2 Maintain Membrane Fluidity in Most Human Cell Types and Independently of Adiponectin — AdipoR1 and AdipoR2 maintain membrane fluidity in most human cell types and independently of adiponectin — Supplemental Data 

# AdipoR1 and AdipoR2 maintain membrane fluidity in most human cell types and independently of adiponectin

## Supplemental Data

- Supplementary Figures (.pdf, 5.9 MB) - Supplementary figures and legends in one combined PDF file.
