# Supplementary material for: AdipoR1 and AdipoR2 maintain membrane fluidity in most human cell types and independently of adiponectin
Source: J Lipid Res. 2019 Mar 19;60(5):995–1004. doi: 10.1194/jlr.M092494 (PMC6495173; doi:10.1194/jlr.M092494)
Supplement: Supplemental Data [file 10.1194_M092494_jlr.M092494-1.pdf]

Supplemental Figures

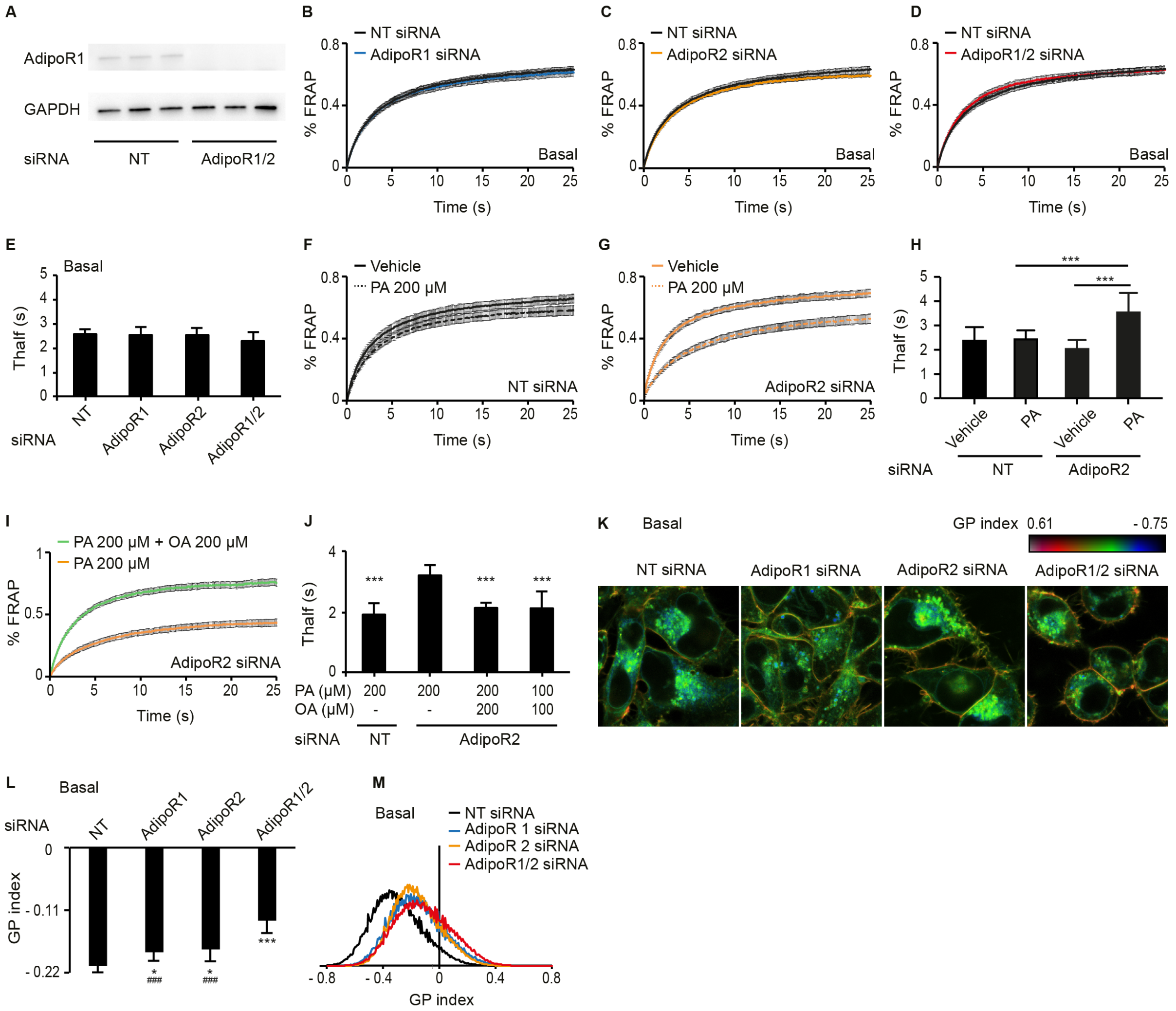

Supplemental Figure S1. FRAP and Laurdan dye analysis of HEK293 cells treated with AdipoR1 and/or AdipoR2 siRNA under basal conditions. (A) Western blot showing downregulation in HEK293 cells treated with siRNA against AdipoR1 and AdipoR2, in independent triplicates. **(B-D)** FRAP results in HEK293 cells treated with NT, AdipoR1, AdipoR2 or a combination of AdipoR1 and AdipoR2 (AdipoR1/2) siRNA (n=6-7). **(E)** Average T_{half} values from B-D. **(F-G)** FRAP results in HEK293 cells treated with vehicle (DMSO) or 200 µM PA and NT siRNA or AdipoR2 siRNA, respectively (n=7-11). **(H)** Average T_{half} values from B-D. **(I)** FRAP result in HEK293 cells treated with PA alone or PA + OA (n=10) **(J)** Average T_{half} values from FRAP experiments with HEK293 cells treated with different amounts of PA and OA and with either NT siRNA or AdipoR2 siRNA (n=10). **(K)** Pseudocolor images showing the Laurdan dye GP index at each pixel position in HEK293 cells treated with NT, AdipoR1 and/or AdipoR2 siRNA. **(L)** Average GP index from several images as in A (n=10). **(M)** Distribution of the GP index values in representative images for each treatment. Error bars show the SD in histograms and SEM in FRAP panels, and significance is indicated as follows: *, ** and *** indicate significant differences from control treatment with p<0.05, 0.01 and 0.001 respectively; # symbols similarly indicate significant differences from the AdipoR1/2 combined siRNA treatment.

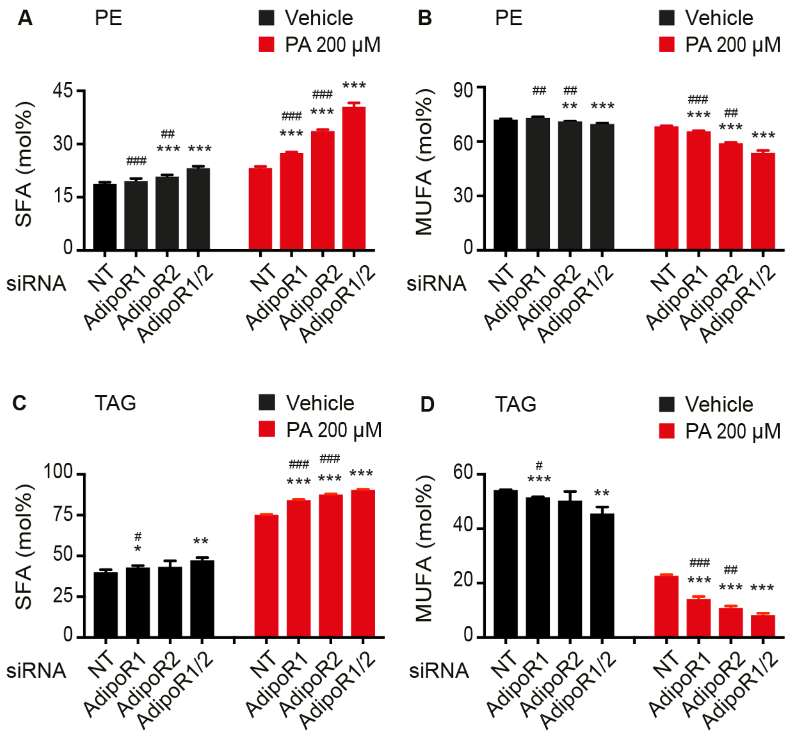

Supplemental Figure S2. The AdipoRs are required to maintain MUFA levels in PEs and TAGs, sustain desaturase gene expression and prevent lipotoxicity by PA in HEK293 cells. (A-D) SFA and MUFA abundance (mol%) in the PEs and TAGs of HEK293 cells cultivated in the presence of either vehicle (DMSO) or 200 µM PA and treated with NT, AdipoR1 and/or AdipoR2 siRNA. Error bars show the SD and significance is indicated as follows: *, ** and *** indicate significant differences from control treatment with p<0.05, 0.01 and 0.001 respectively; # symbols similarly indicate significant differences from the AdipoR1/2 combined siRNA treatment.

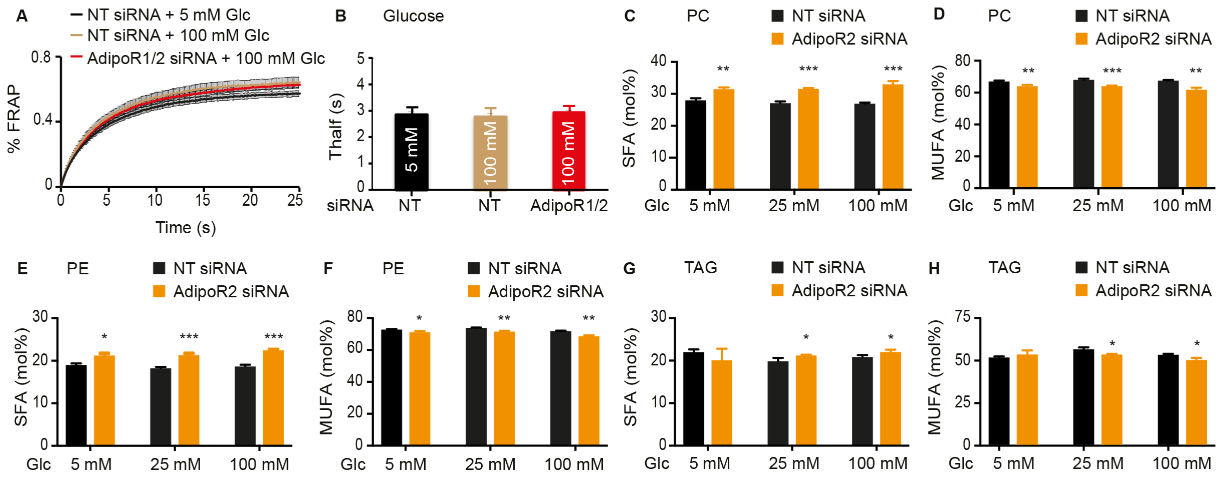

Supplemental Figure S3. Glucose does not affect membrane fluidity or composition in HEK293 cells. (A) FRAP results in HEK293 cells cultivated in 5 mM glucose or challenged with 100 mM glucose and treated with NT or combined AdipoR1/2 siRNA (n=5-7). **(B)** Average T_{half} values (the time by which half of the maximum fluorescence recovery is reached) from A. **(C-H)** SFA and MUFA abundance (mol%) in the PCs, PEs and TAGs of HEK293 cells cultivated in 5 mM glucose or challenged with 25 mM or 100 mM glucose and treated with NT or combined AdipoR1/2 siRNA (n=3). Error bars show the SD in histograms and SEM in the FRAP panel, and significance is indicated as follows: *, ** and *** indicate significant differences from control treatment with p<0.05, 0.01 and 0.001 respectively.

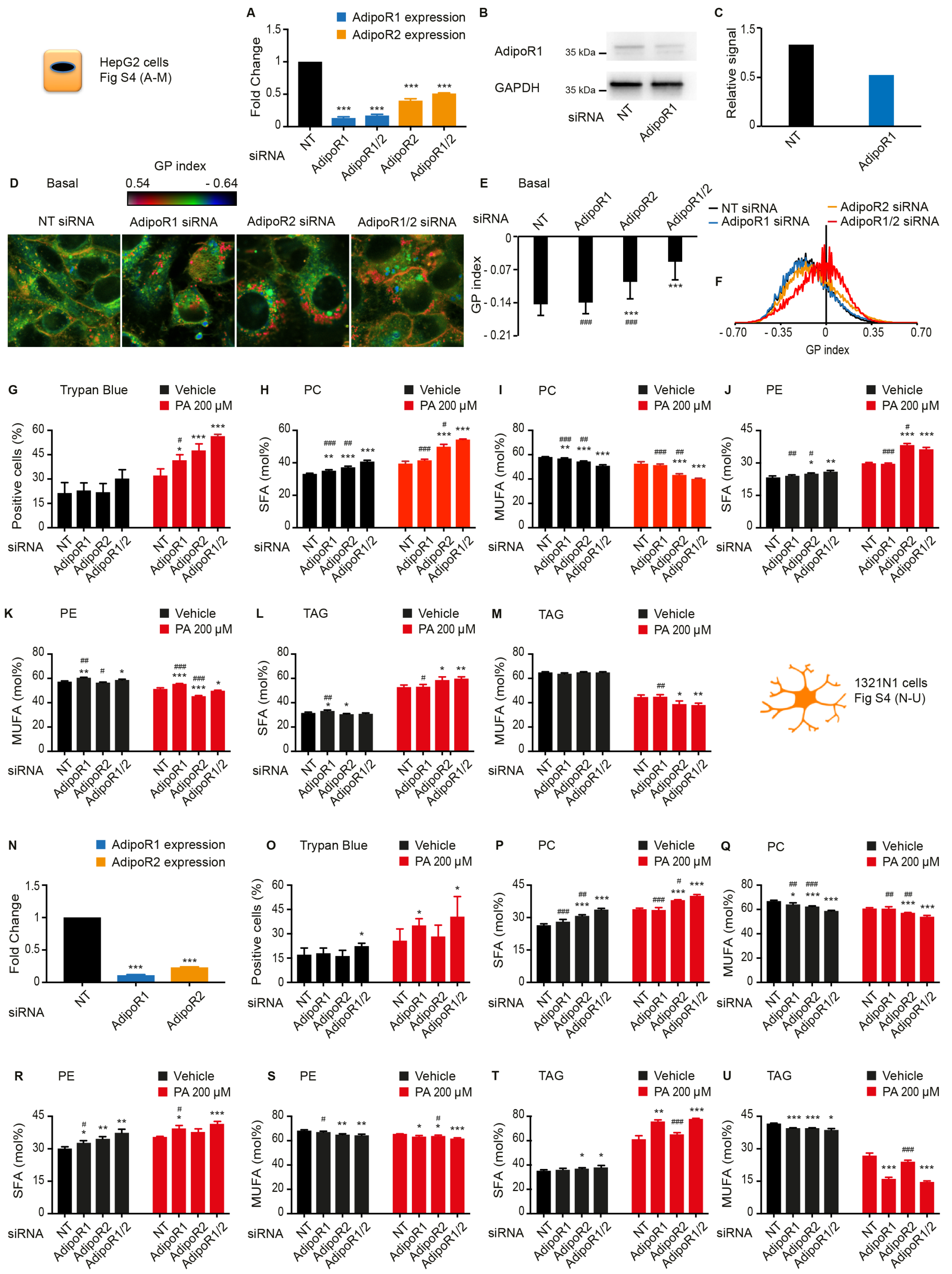

Supplemental Figure S4. The AdipoRs are required to maintain membrane fluidity and MUFA levels, and prevent lipotoxicity in HepG2 and 1321N1 cells. (A) qPCR results showing the efficiency of the knockdown using NT, AdipoR1 and/or AdipoR2 siRNA in HepG2 cells. The expression levels

are normalized to the NT value. **(B-C)** Western blot and quantification, respectively, to detect AdipoR1 protein in HepG2 cells treated with NT siRNA or AdipoR1 siRNA; GAPDH is used as a loading control. **(D)** Pseudocolor images showing the Laurdan dye GP index at each pixel position in HepG2 cells treated with NT, AdipoR1 and/or AdipoR2 siRNA. **(E)** Average GP index from several images as in B (n=14-15). **(F)** Distribution of the GP index values in representative images for each treatment from B. **(G)** Percentage of dead HepG2 cells (Trypan Blue-positive) following cultivation in the presence of either vehicle (DMSO) or 200 µM PA and treated with NT, AdipoR1 and/or AdipoR2 siRNA. **(H-M)** SFA and MUFA abundance (mol%) in the PCs, PEs and TAGs of HepG2 cells cultivated in the presence of either vehicle (DMSO) or 200 µM PA and treated with NT, AdipoR1 and/or AdipoR2 siRNA (n=3). **(N)** qPCR results showing the efficiency of the knockdown using NT, AdipoR1 and/or AdipoR2 siRNA in 1321N1 cells. The expression levels are normalized to the NT value. **(O)** Percentage of dead 1321N1 cells (Trypan Blue-positive) following cultivation in the presence of either vehicle (DMSO) or 200 µM PA and treated with NT, AdipoR1 and/or AdipoR2 siRNA. **(P-U)** SFA and MUFA abundance (mol%) in the PCs, PEs and TAGs of 1321N cells cultivated in the presence of either vehicle (DMSO) or 200 µM PA and treated with NT, AdipoR1 and/or AdipoR2 siRNA (n=3). Error bars show the SD and significance is indicated as follows: *, ** and *** indicate significant differences from control treatment with $p<0.05$, 0.01 and 0.001 respectively; # symbols similarly indicate significant differences from the AdipoR1/2 combined siRNA treatment.

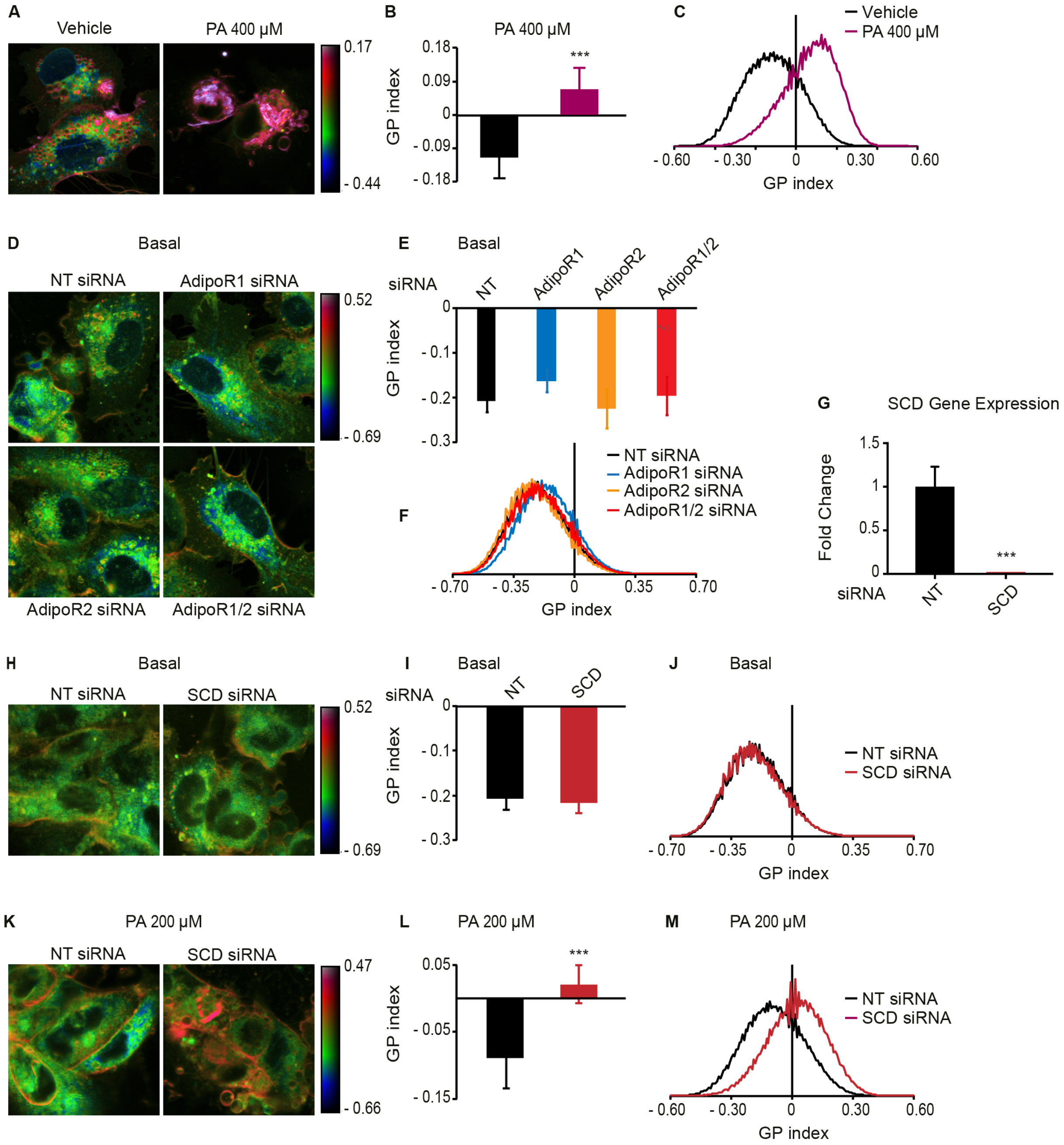

Supplemental Figure S5. Membrane fluidity of HUVEC cells in high palmitate, in basal conditions or following knockdown of the AdipoRs or SCD. (A) Pseudocolor images showing the Laurdan dye GP index at each pixel position in HUVEC cells treated with vehicle (DMSO) or 400 μM PA. **(B)** Average GP index from several images as in B (n=15). **(C)** Distribution of the GP index values in representative images for each treatment from B. **(D)** False color images showing the Laurdan dye GP index at each pixel position in HUVEC cells cultivated in basal conditions and treated with NT, AdipoR1 and/or AdipoR2 siRNA. **(E)** Average GP index from several images as in D (n=9-10). **(F)** Distribution of the GP index values in representative images for each treatment from D. **(G)** qPCR results showing the efficiency of the SCD knockdown using NT or SCD siRNA in HUVEC cells. The expression levels are normalized to the NT value. **(H)** Pseudocolor images showing the Laurdan dye GP index at each pixel position in HUVEC cells cultivated in basal conditions and treated with NT or SCD siRNA. **(I)**

Average GP index from several images as in H (n=9-10). **(J)** Distribution of the GP index values in representative images for each treatment from H. **(K)** Pseudocolor images showing the Laurdan dye GP index at each pixel position in HUVEC cells cultivated in the presence of 200 μM PA and treated with NT or SCD siRNA. **(L)** Average GP index from several images as in H (n=15). **(M)** Distribution of the GP index values in representative images for each treatment from H. Error bars show the SD and significance is indicated as follows: *, ** and *** indicate significant differences from control treatment with $p<0.05$, 0.01 and 0.001 respectively; # symbols similarly indicate significant differences from the AdipoR1/2 combined siRNA treatment.

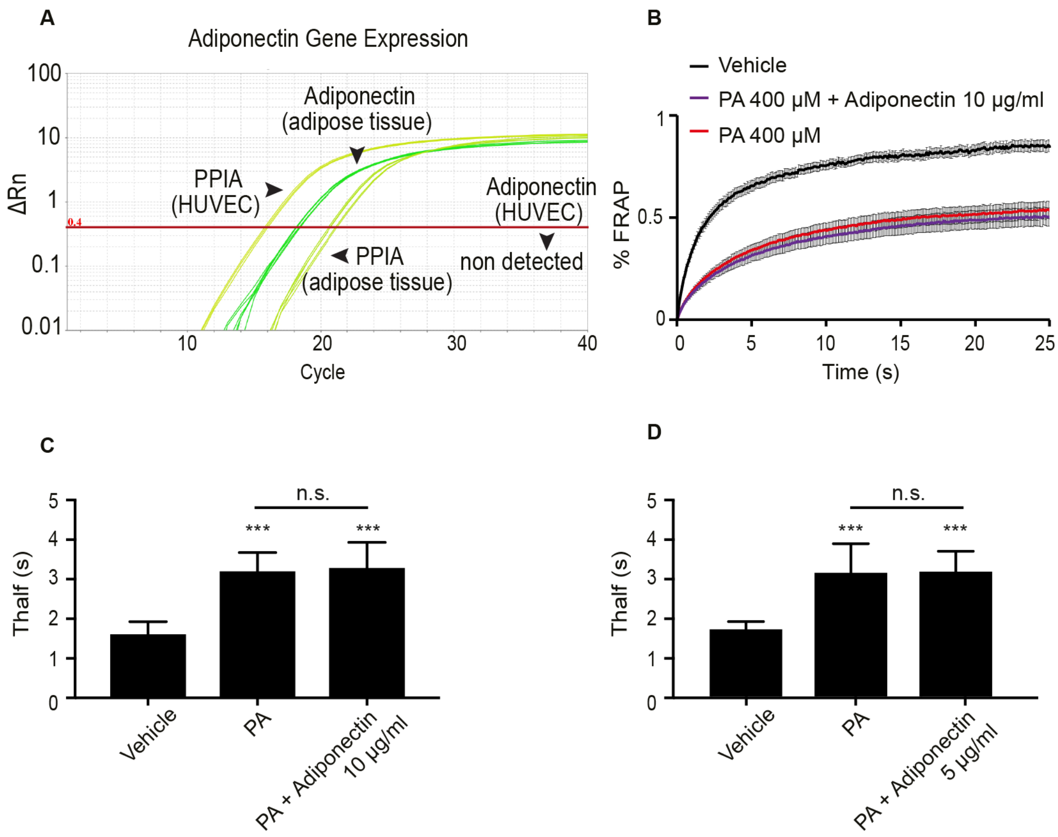

Supplemental Figure S6. The cell types studied do not express the adiponectin gene and adiponectin does not improve membrane homeostasis in HEK293 cells. **(A)** qPCR intensity curves showing that human adipose tissue expresses high levels of the adiponectin (aka AdipoQ) gene, reaching the normalized reference value ΔRn 0.4 by cycle 19 (red line). No adiponectin expression was detected in HUVEC cells. Both adipose tissue and HUVEC cells express high levels of the house-keeping gene PPIA. **(B)** FRAP result showing that addition of 10 µg/ml of adiponectin does not improve membrane fluidity in HEK293 cells challenged with 400 µM PA. **(C** and **D)** Average T_{half} values from FRAP experiments using 10 µg /ml Adiponectin (n=9-11) or 5 µg /ml Adiponectin (n=6 for vehicle and n=12-14 for the two other conditions). Error bars show the SD in histograms and SEM in the FRAP panel, and *** indicate significant differences from control treatment with p<0.001.
